# Patients’ perception of quality of nursing care; a tertiary center experience from Ethiopia

**DOI:** 10.1186/s12912-019-0361-z

**Published:** 2019-08-14

**Authors:** Teshome Gishu, Abate Yeshidinber Weldetsadik, Atnafu Mekonnen Tekleab

**Affiliations:** 1 School of Nursing, Saint Paul’s Hospital Millennium Medical College (SPHMMC), Addis Ababa, Ethiopia; 2 Department of Pediatrics and Child Health, Saint Paul’s Hospital Millennium Medical College (SPHMMC), P.O. Box 1271, Addis Ababa, Ethiopia

**Keywords:** Quality-nursing care, Perception, Patient, Ethiopia

## Abstract

**Background:**

Nursing care closely influences patients’ satisfaction with the overall quality of care, and the importance of measuring patient satisfaction with nursing care cannot be emphasized enough. Data are however scarce regarding patients’ perception of quality of nursing care in Ethiopia. We performed this study to assess patient’s perception of the quality of nursing care in a tertiary center in Ethiopia.

**Methods:**

Data were collected prospectively using Quality of Nursing Care Questionnaires-patient of Safford & Schlotfeldt. A total of 340 patients were included using systematic random sampling and data were analyzed using SPSS for windows version- 20.

**Result:**

The nursing care performance was highest for nurse-physician relation (mean = 3.95) and low for education and home care preparation and physical care (mean score of 2.79 and 2.89 respectively). The emotional care and nurse administration mean score were 3.5 and 3.83 respectively. The overall nursing quality was neither satisfying nor dissatisfying (mean of 3.39). While only 36% of the respondents were satisfied with the nursing care, patient education has the strongest (AOR of 7.4) association with satisfaction.

**Conclusion:**

Patients perceived low quality of physical care, education and preparation for home care but better nurse-physician relation and nursing administration. However the overall quality measure was neither satisfying nor dissatisfying. This calls for an action from the health care administrators, educators and other stakeholders to improve the patient perception of quality nursing care.

## Background

Patients’ perception of care quality refers to patients’ view of services received and the results of the treatment and are monitored to assess the delivery and quality of healthcare, while patient experiences is a reflection of what actually happened during the care process [1, 2]. Improving the quality of health care delivery is an important global priority and the purpose of health care quality improvement initiatives is to ensure patient safety, improve clinical effectiveness, and promote public accountability [3–5].

Providing high-quality care and ensuring patient satisfaction is a challenge that healthcare organizations face globally. Exploring the quality of nursing care from the patients’ perspective including patient satisfaction has been an essential part of quality of health care evaluation. As a result, hospital management and accreditations require regular measurement of patient satisfaction and experience as integral part of their quality evaluation process despite its complexity and difficulty to measure [6–10].

Patient satisfaction is the link between their perceptions of quality and their future intention to use the service or recommend it to others. As patient satisfaction is an important indicator of nursing and overall quality of care, a more focused and direct measurement of patient perception of nursing quality care is warranted and some validated tools have been developed specifically to assess the patient perception of quality of care [11–14]. Nursing care most closely influences patients’ satisfaction with the overall quality of care, and therefore, the importance of measuring patient perception of quality of nursing care cannot be emphasized enough [10, 15]. In line with this, a recent Australian study demonstrated the direct relation of patient experience and perception of nursing quality of care [16].

Quality of nursing care has been among the major focus for the public and the Ethiopian government and the federal ministry of health has been running a sector wide reform aimed at improving the quality and accessibility of health service at all levels of the country through implementing hospital reform guideline; one of the main components of this guideline focusing on improving quality of care [15–17, 18]. Patient perception of quality of care is inadequately explored especially in developing countries including Ethiopia. Assessing patient perception and experiences of the quality of care not only provides information about the actual experiences, but also reveals which quality aspects patients regard as most important [12, 19]. Despite presence of many studies of the determinants and status of patient perception on quality of nursing care worldwide, there is scarcity of evidences in Ethiopia inspiring us to undergo this study to assess the perception of patients on the quality of nursing care in St Paul’s Hospital Millennium Medical College, one of the biggest tertiary centers in the country.

## Methods

### Setting

The study was conducted in SPHMMC departments of pediatrics and child health, obstetrics/gynecology, internal medicine and surgery. The annual patient load of the hospital is more than 300,000 and mostly serves underprivileged patients with poor socioeconomic status and more than 80% of the clients don’t afford to pay for their health care and are served free of charge. There are more than 1500 health care professionals working at SPHMMC, most (53%) of whom are nurses with BSc in nursing. The nurses are responsible for clinical care, patient and family education, quality assurance and also take part in administrative activities in their respective clinics and wards.

### Study design and period

Cross-sectional descriptive study was conducted over a period of 6 weeks from March 1, 2016 to April 4, 2016.

### Sample size determination

The number of patients surveyed was calculated using the single proportion population formula z **=** z^2^ p (1-p)/d^2^. Taking 32.9% (18) prevalence of patient dissatisfaction with the quality of the nursing care, 95% confidence interval (CI) and 5% margin of error, the calculated sample size were 340 patients. Where: N = sample size determined (340), Z = 95% confidence interval (1.96), P = prevalence of patient dissatisfaction in hospital (32.9%) and D = margin of error (5%).

### Measurement

Quality of Nursing Care Questionnaires-Patient of Safford & Schlotfeldt [20] with a 5 point Likert scale (rated as: 1 = never, 2 = seldom, 3 = sometimes, 4 = usually, 5 = always) was used to measure patients’ perception of quality of nursing care. Quality of Nursing Care Questionnaires-Patient has 43 items organized in five subscales: physical care (12 items), emotional care (17 items), nurse physician team work (1 item), preparation for home care (7 items), and nursing administration (6 items). A sixth variable; quality of nursing care, is calculated as a composite score of the other five subscales. The mean and standard deviations of each subscale is calculated to describe the result, and the average mean of the five subscales is used to calculate the quality of nursing care. A mean score of ≥4 shows the quality of care is satisfying, 2.5–4 is neither satisfying nor dissatisfying and < 2.5 is dissatisfying. The questionnaire (instrument) was selected based on the model of nursing care used in our hospital which uses all the above five subscales as center of nursing care practice. Translation to local language was done by the research team supported by a linguistic expert and translation back to English was done to confirm consistency. Minor modifications were done to the local context after the team discussion and pilot testing of the questionnaire. Included within these subscales of nursing care models are the different specific variables that are associated with the satisfaction of patients and we analyzed them using multivariate logistic regression to see which of these factors are regarded by our patient populations as more important indicators of quality of nursing care.

### Sampling technique and data collection procedure

Patients included in the study had to stay seven days in the ward so that they could have adequate experience with all the nurses working in the wards as nurse’s shift is every other day in four groups in most of the departments. A stratified random sampling technique was employed based on the admission rate to the different departments in the preceding three months before the study period to recruit patients who were included in the study. The sampling frame was the bed number of each ward. After the sample was determined for each department, Patients who were occupying beds of every other number was included in the study. The first bed of the sampling was selected based on simple random sampling technique from the first two beds in each ward. Once a patient who had been included in the study was discharged, the next patient who was admitted in the same bed was recruited for the study. The data collection was continued until the calculated sample size was reached.

Once a patient was recruited in the study, data collectors approached the patient. Data was collected by interviewing the patient using paper based questionnaire at the bedside. In the department of pediatrics the respondents were the parents or care takers of the child who had spent with the child most of the time while the child was in the hospital. The questionnaire was translated from English language to the local Amharic and Afan Oromo languages and back to English. Previous psychometric property was not available for the instrument but the translated questionnaire has very good reliability with Cronbach’s alpha of 0.9(0.83–0.93).

Data was collected by two trained BSC nurses who are not working in the hospital with the intension of decreasing biases. Both data collectors were fluent speakers of the two most commonly spoken local languages. The data collectors were trained on questionnaire content, interviewing technique and operational definitions of the research.

### Eligible criteria

Patients who had been in the ward for at least seven days at the time of the data collection were included in the study and patient who is critically sick or unconscious or who had mental illness were excluded after observation of their medical record and evaluation by the research nurses directly. Mental illness was defined as any form of current psychiatric illness diagnosed and documented by physician in the patients’ medical record during the current admission.

### Data analysis

Data was analyzed using SPSS for windows version 21.0. Data was cleaned and checked for consistency and completeness and no missing values were found. Descriptive analysis was done by running simple frequencies and proportions. The information obtained from the patients was used to calculate the mean score of each subscale and a final mean for quality of nursing care to evaluate the quality nursing care. Binary logistic regression was used to assess predictors of patient satisfaction and *p* value less than 0.05 were considered significant. Variables in each subscales with a p value of < 0.1 were entered in multivariate analysis to see independent factors associated with patient satisfaction. We didn’t look in to differences in the different units however.

### Ethical consideration

The study was conducted after obtaining ethical clearance from the Institutional Review Board/IRB of SPHMMC. Consent was obtained from the patients before the interview and confidentiality was assured. There was no risk or harm to the participant and they had the right not to participate if they wanted and no personal identifiers were used.

## Result

A total of 340 patients were included in our study. Patients included were different group of population with any disease requiring admission to the departments of Pediatrics (care givers satisfaction taken as client satisfaction), Obstetrics and gynecology, Internal medicine and Surgery with nearly equal distribution from each unit. Among the 340 patients, only 125 (36.8%) experienced high overall satisfaction (perceived high quality of nursing care). The mean quality of nursing care score was 3.39 which is neither satisfying nor dissatisfying. The highest mean was for Nurse Physician relationship (mean = 3.95) but lowest for teaching and preparation for home care and physical care (Table 1). On assessment of factors predicting overall satisfaction, components of physical care, emotional care, patient education and nurse physician team work were significant determinants of patient satisfaction, but no association was found between nursing administrative responsibilities and patient satisfaction (Table 2). On multivariate logistic regression nurse having adequate time, patient comfort, explaining what happen to patient, nurses’ –physician team work and educating patient how to self-care are associated with better satisfaction. Among this patient education how to self-care has the highest association with AOR of 7.40(3.96–13.82) (Table 2).

**Table 1:** Mean and standard deviation (SD) of nursing care parameters assessed in the study

Subscale	Mean score	SD
Physical care	2.89	1.16
Emotional care	3.50	1.10
Nurse physician relationship	3.95	0.95
Teaching and preparation for home care	2.79	1.63
Nursing administration	3.83	0.93
Quality of nursing care	3.39	–

**Table 2:** Multivariate analysis of selected variables affecting patient satisfaction level

Variables	p value	AOR	95% C. I for EXP(B)	
			Lower	Upper
Had adequate time for your care	.001	1.82	1.27	2.62
Keep your comfort	<.0001	1.82	1.33	2.48
Nurse appear feeling good	.600	0.90	0.62	1.32
Explain what happen to you	.004	1.75	1.20	2.57
Keep your room neat	.350	0.82	0.55	1.24
Nurse physician team work	.010	1.71	1.10	2.68
Teach you how to self-care	<.0001	7.40	3.96	13.82

*SD* Standard deviation

### Physical care

Patients perceived low quality of physical care with a mean score of 2.89. The exception was for nurses carrying out treatments and medications on time with 326(95.8%) of the patients being satisfied. The lowest performance is for oral care and patient bath with only 18.3 and 9.7% satisfaction rates respectively. Nearly a third of patients (117(34.4%)) never get nurses help in getting in and out of bed and only 186(54.7%) of patient calls received nurses answer promptly.

### Emotional care

About 103 (30.3%) patients felt that the nurses are never or seldom informed of their needs. The highest score is for nurses keep the room neat satisfying 73% of patients. Only half of patients’ religious needs were attended by their nurses regularly. One-hundred-eighty three (53%) of the patients felt confidence in their nurses usually or always. On the other hand, 192(56.5%) of patients were satisfied by nurses respectful care. A significant number of patients (111(32.6%)) felt that nurses were interested in their patient as well as their patients welfare only sometimes.

### Nurse physician relationship

Despite having the highest mean (3.95), only two hundred and forty two patients (71.2%) reported that their nurses understand the physicians plan for their care making the team work neither satisfying nor dissatisfying overall.

### Teaching and preparation for home care

While patient education is least performed by our nurses (mean of 2.79), it is perceived by patients as the most important part of the care (Table 2). However, only a third of families and patients (108(31.8%) and 106(31.2%) respectively) were given adequate education on home care. Education was not given at all to 188(55.3%) patients and 136(40.0%) of their families.

### Nursing administration

Most (74.2%) patients were satisfied with availability of supplies and equipment needed to give good care. Two hundred and forty two (70.8%) of them reported receiving enough attention from their nurse usually or always.

*AOR* adjusted odds ratio

## Discussion

We evaluated perception of quality of nursing care in 340 patients among whom only 125 (36.8%) were satisfied with the quality of nursing care. The remaining majority were dissatisfied or neither satisfied nor dissatisfied. Such a low perception of quality of care by patients may create problem in the health system by decreasing trust and less utilization of services as well as delayed health seeking with subsequent poor health outcome of the public. This is especially important in developing countries as demonstrated from a previous study in Bangladesh [21]. Hospital administrators, nurse educators and health authorities of the country should emphasize on efforts that maximize patients’ positive perception of quality of nursing care [22–27].

Four of the nursing care components (but not nursing administration) were found to affect patient satisfaction in our study significantly. While it is well documented that nursing administration also affects the quality of care, it is likely to have an indirect effect not easily visible to our clients as the other subscales which are directly observable [28, 29].

Among the five components of quality nursing care measures the highest mean score was for Nurse Physician relationship (mean = 3.95). Despite the highest score, however, it was documented as “always” only in a third of cases (33%) which means that even the best performance is far from the patients’ expectation. In a recent study from the same setting, the nurse physician relation was perceived to be the lowest of all parameters by both physicians and nurses in contrary to the patients’ perception [28]. However, the patient perception questionnaire was represented only with a single item and is less exhaustive than the physician and nurse perception questionnaires [20]. The other possible explanation might be lesser understanding of patients as the hospital serves mostly underprivileged population in the region [30, 31]. On the other hand, this observation might be reflection of the different understanding of the important quality of care measures among health care providers and receivers. It has also been documented from a recent study that perception of quality of care between physicians and nurses is different [28, 32, 33].

Physical care and patient education were among the least performed with mean scores of 2.89 and 2.79 respectively. In line with this, education was found to be one of the inadequately performed nurses’ job in a large multicenter European study as high as in 41% of the cases while it was never done in 55.3% in our study [34]. The low nursing performance in patient education on one side and the strong association between patient education and satisfaction on the other side (AOR of 7) calls for action that the hospital administrators and other responsible bodies should enforce to implement these undone nursing activities regularly.

Despite a better mean score of the emotional care and nursing administration, patients perceived low quality of care in both subscales. Some examples of this includes nurses not well informed of patient needs (61.5%), inadequate nursing team work (31.2%), nurses not knowledge of their job (33.9%), nurses not interested in patient welfare (51.7%), nurses don’t have time to take care patients (43.2) and nurses don’t attend to patient religious needs (49.4%). It is however important to notice that these figures showed unmet needs of patients with no further information in the contribution of each aspect. Even if we use all of them to calculate the composite score of quality of care, this may not be practically similar for individual patients as different patients will have different priorities and needs.

The overall patient perceived quality of nursing care is neither satisfying nor dissatisfying in our study. The result is lower than a recent Ethiopian [35] and Jordanian [29] studies but comparable with a previous Ethiopian study from Jimma [30]. The quality of care was also perceived to be low by health care providers from a study in the same setting [28]. In addition to the different design, instruments and working environment; the difference of the satisfaction rate is likely the reflection of which quality aspects patients regard as most important and also deficient as is patient education in our study [36–39, 28]. Our finding also strengthen observations that factors responsible for rate of patient satisfaction and quality of care differs in different settings and regions of the world [21, 40]. The implication of such studies goes beyond hospital administrators as the evidences enforce collaboration among health authorities, nurse educators, researchers, professional societies, the public and other stake holders to achieve the maximum possible result.

### Limitations

Our study was done in a single center making generalization to a national level difficult and we didn’t look the difference in the different units despite the system and nursing environment similarity. The open bedside interview we use to collect data might also influence the participants’ response to some extent even if the data collectors were not part of the treating team.

## Conclusion

Patients perceived low quality of physical care, education and preparation for home care but better nurse physician relation and nursing administration in our study. The overall quality of nursing care was however neither satisfying nor dissatisfying. This calls for an action from the health care stakeholders to improve quality of nursing care and patient satisfaction.

## Data Availability

Data are available with the authors for review as necessary.

## Abbreviations

CI: Confidence interval
IRB: Institutional review board
OR: Odds ratio
SPHMMC: St Paul’s hospital millennium medical college
